# Proteome analysis refines molecular processes underlying metamorphosis in the ascidian *Ciona intestinalis*

**DOI:** 10.1371/journal.pone.0350646

**Published:** 2026-06-01

**Authors:** Daniele Capitanio, Silvia Mercurio, Ettore Mosca, Cristina Battaglia, Marco Venturin, Roberta Pennati

**Affiliations:** 1 Department of Biomedical Sciences for Health, Università degli Studi di Milano, Milan, Italy; 2 Department of Environmental Science and Policy, Università degli Studi di Milano, Milan, Italy; 3 Institute of Biomedical Technologies, National Research Council, Segrate, Italy; 4 Department of Medical Biotechnology and Translational Medicine, Università degli Studi di Milano, Milan, Italy; Academia Sinica, TAIWAN

## Abstract

Tunicates, including ascidians, are recognized as the true ‘sister group’ of vertebrates and are emerging as models to study larval and post-larval development, including degeneration of central nervous system (CNS), in chordates. Ascidian larvae have the typical chordate body plan that includes a dorsal neural tube. During their metamorphosis, a deep tissue reorganization takes place, with some tissues that degenerate while others develop to become functional during the adult life. The larval CNS also degenerates, and most neurons disappear, making room for the formation of adult CNS. The genome of the ascidian *Ciona intestinalis* has been sequenced and annotated, with several CNS specific genes that have been characterized. These features make ascidian metamorphosis a good model to study the mechanisms underlying physiological CNS degeneration. To shed light on the molecular determinants of *C. intestinalis* metamorphosis, we analyzed the proteome at three stages of development: swimming larva (SwL, Hotta stage 28), settled larva (SetL, Hotta stage 32) and metamorphosing larva (MetL, Hotta stage 34). A total of 405 modulated proteins were identified by mass spectrometry by comparing the three stages. Enrichment and network analysis showed the involvement of several processes/pathways, including autophagy and mTOR pathways, and actin cytoskeleton organization and remodeling among the most significant ones. This study helps to elucidate the molecular processes underlying ascidian metamorphosis and provides insight into mechanisms of physiological neurodegeneration in ascidians.

## Introduction

Tunicates have been recognized as the sister group of vertebrates with which they share an exclusive common ancestor [1] and are emerging as excellent models to study embryonic and post-larval development, aging and degeneration of central nervous system (CNS) [2,3]. Ascidians are the most numerous group of tunicates; they are sessile filter feeder animals that develop through a swimming lecithotrophic tadpole larva. The larva retains the main key characters of the chordate body plan including a dorsal hollow neural tube that lays dorsal to the notochord [4]. The larval CNS of *Ciona intestinalis* (here after *Ciona*), one of the most studied ascidians, consists of less than 100 neurons and 250 glial cells [5]. It contains suitably few cells to enable the final counts, their identification, the developmental history and the analysis of functions cell by cell [6]. The CNS can be subdivided morphologically and functionally along the anteroposterior axis into four regions similarly to the vertebrate one: the sensory vesicle with the sensory organs, the neck, the visceral ganglion and the tail nerve cord. Besides its small size, it includes several neuronal types, distinguishable by the neurotransmitter that they utilize [7]. Moreover, the CNS of ascidians develops through a process of neurulation that takes place at the end of the gastrula period and starts with the differentiation of a neural plate [8].

After a short swimming period, the ascidian larva undergoes a deep morphological change and becomes sessile. The metamorphic process is characterized by a rearrangement of larval tissue and organs, some of which degenerate and are reabsorbed, whereas others develop and become functional for adult life [9]. Most larval neurons, except for a few motor neurons and glutamatergic neurons, disappear during the process of adult CNS formation, whereas some larval ependymal cells differentiate into neurons and contribute to the construction of adult CNS [10]. The metamorphosis in ascidians is so extreme, and the adult body so derived that, before the larval stage was known, ascidians were not even classified as chordates. Besides species-specific traits, metamorphosis in all ascidians is characterized by ten events that were described by Cloney [11] and recently reviewed [12], starting with the secretion of adhesives by the papillae or epidermis of the trunk, and terminating with the release of organ rudiments from an arrested state of development. These drastic structural changes are driven by several molecular processes including apoptosis [13], cell proliferation and differentiation [14], cell migration [15]. These characteristics make the ascidian metamorphosis an exceptional model to study the mechanisms that regulate metamorphosis in animals and neurodegeneration in physiological conditions.

The genome of *Ciona* was published in 2002 [16]. It is considered quite simple since the size is approximately 160 mega base pairs (Mbp) per haploid and contains approximately 16000 genes. Since the genome was published, its wide analyses have successfully characterized an increasing number of genes specifically expressed during the metamorphosis [17]. Nevertheless, proteomic studies are still scarce. In this paper, we compared the proteomes of swimming larvae, individuals at the onset of metamorphosis, and newly metamorphosed juveniles to identify differentially expressed proteins, focusing on proteins known to play a role in nervous system development and functioning.

## Materials and methods

### Animals

Adults of *Ciona intestinalis* were collected by the fishery service of the Station Biologique de Roscoff (France). In Italy, the use of invertebrates — except for cephalopods — is not covered by current legislation (Legislative Decree no. 26/2014). Therefore, laboratory experiments could be conducted without requiring prior authorization. Once in the laboratory, animals were maintained in aquaria at 18°C for two days for acclimatization. To obtain synchronously developing embryos, in vitro fertilization was performed with gametes collected by dissection from five adults. Embryos were reared at 18°C in artificial seawater supplemented with 0.1 mM HEPES (Sigma, Italy) (AFWH) until they reached the larva stage. Since after hatching the development is no longer synchronous, hatched larvae were carefully monitored by a stereo microscope to identify individuals that reached the selected developmental stages. When samples reached the desired developmental stage, they were manually collected, pooled in groups of 200 individuals, and immediately frozen in liquid nitrogen. The fertilization was replicated three times to obtain three biological replicas.

To catch the molecular events of neurodegeneration, we selected three developmental stages: i) swimming larva stage (SwL, stage 28 of Hotta) [9], characterized by elongated papillae and a square shaped trunk. The larvae were actively swimming with vigorous tail movements. This stage was reached about 2 hours post hatching; ii) settled larva (SetL corresponding to Mid Tail Absorption Stage 32 of Hotta), the larva was attached to the substrate by means of the adhesive papillae. The tail was not moving and at least 50% of the tail was resorbed into the larval trunk. This stage was reached 10–11 hours after hatching; iii) metamorphosed larva (MetL, corresponding to the Early Body Axis Rotation Stage 34 of Hotta), the stalk was elongated and formed an angle of about 90° with the endostyle axis. This stage was reached 14–18 hours after hatching.

### Protein extraction and proteomic analysis

A total of 9 samples were collected from three stages of larval development of *Ciona* (3 biological replicates for each stage) and their proteins were extracted in lysis buffer (0.1 M Tris/HCl pH 7.6, 4% SDS, 0.1 M DTT and 0.001 M PMSF) through sonication. Proteins were denatured at 95°C for 3 min and lysates clarified by centrifugation at 16000 g for 15 min. Protein concentrations were determined using the 2D-Quant Kit (Cytiva) and 120 µg of each extract were processed following the Filter aided sample preparation (FASP) method [18]. Then liquid chromatography coupled to electrospray tandem mass spectrometry (LC–ESI–MS/MS) with label-free quantification analysis was performed. All samples were analyzed in triplicate at UNITECH OMICs (University of Milano, Italy) using a Dionex Ultimate 3000 nano-LC system (Sunnyvale CA, USA) connected to an Orbitrap Fusion™ Tribrid™ Mass Spectrometer (Thermo Scientific, Bremen, Germany) equipped with nano electrospray ion source. Peptide mixtures were pre-concentrated onto an Acclaim PepMap 100 – 100 μm x 2 cm C18 (Thermo Scientific) and separated on EASY-Spray column ES802A, 25 cm x 75 μm ID packed with Thermo Scientific Acclaim PepMap RSLC C18, 3 μm, 100 Å using mobile phase A (0,1% formic acid in water) and mobile phase B (0.1% formic acid in acetonitrile 20/80, v/v) at a flow rate of 0.300 μL/min. The temperature was set to 35°C. The sample injection volume was 3 μL. MS spectra were collected over an m/z range of 375–1500 Da at 120,000 resolutions, operating in the data dependent mode (TOP 40). HCD was performed with collision energy set at 35 eV. Polarity: positive.

### Bioinformatics and statistical analysis

#### Protein annotation and differential expression analysis.

Proteins were identified to match with *Ciona intestinalis* UNIPROT reference proteome (UP000008144, https://www.uniprot.org/proteomes/UP000008144) and their peaks were quantified with MaxQuant software (version 2.7.0.0, Max Planck Institute of Biochemistry, Germany) [19]. Quantification in MaxQuant was performed using the built-in extracted ion chromatogram (XIC)-based label-free quantification (LFQ) algorithm using fast LFQ [20]. Detection of differentially expressed proteins (DEPs) was performed with Perseus software [21] (version 2.0.11, Max Planck Institute of Biochemistry). ANOVA and Tukey post-hoc test (p-value < 0.05) were used to obtain DE proteins with their corresponding log fold change (LFC) values for the pairwise comparisons between the three developmental stages. Proteins with less than 6 valid values in at least 1 group were filtered out. False positives were excluded utilizing the Benjamini–Hochberg false discovery rate test. STRING (Version 12.0, https://string-db.org/) annotation was used to further characterize DE proteins [22]. Venn diagram was generated using the jvenn web application (https://jvenn.toulouse.inrae.fr/app/index.html).

The selection of proteins defined as ‘neuronal’ was performed manually by filtering the functional annotations produced by STRING after querying the Gene Ontology databases (Biological Process and Cellular Component), Reactome, InterPro, and STRING clusters for the terms ‘neuron*’ and ‘nerv*’. The comparison with the transcriptome profile of swimming larva central nervous system published in [23] was carried out by downloading the ‘KH2012 vs Ensembl’ file from the Aniseed website (https://www.aniseed.fr/), to convert *Ciona* KH2012 gene IDs into the corresponding Ensembl IDs.

#### Pathway and gene ontology enrichment analysis.

Functional enrichment analysis was performed with the g:GOSt tool of the g:Profiler web server (https://biit.cs.ut.ee/gprofiler/gost), using default parameters. The enrichment bubble plots were generated using the online SRplot tool (https://bioinformatics.com.cn) and edited with the Adobe Illustrator CS3 software (version 13.0.0).

#### Protein-protein interaction (PPI) network analysis.

Network analysis of differentially expressed proteins across the three comparisons was performed using STRING, applying the full network type and a minimum required interaction score of 0.7 (high confidence). Network clustering was performed using the modularity optimization algorithms “multi-level” [24] and “fast greedy” [25], available in the R package igraph (https://doi.org/10.48550/arXiv.2311.10260). The functional cartography was performed using the network analysis tool dmfind v2 [26], available at the URL https://github.com/emosca-cnr/dmfind. The participation coefficient (P) and the within-module (community) degree z-score (z) of a protein i were defined as:


$$\begin{array}{c} \begin{array}{cc} P_{i}=1-\sum_{j=1}^{n}\left(\frac{k_{ij}}{K_{i}}\right), & z_{i}=\;\frac{k_{ij}-m_{j}}{\sigma_{j}}, \end{array} \end{array}$$

where *n* is the number of communities, $K_{i}\;$is the number of interactions of protein i, $k_{ij}$ is the number of interactions of protein i within community j, $m_{j}$ is the average $k_{ij}$ over all proteins of the community j, and $\sigma_{j}$ is the standard deviation of $k_{ij}$ over all proteins of the community j.

The over representation analysis on network communities was performed using protein GO biological process (BP) terms provided by STRING (v12) [22]. Only GO BP terms containing at most 500 proteins and at least 3 proteins of the same network community were considered. The reference universe for the hypergeometric test was defined by all the proteins of the network under analysis.

The processed networks files were imported into Cytoscape (version 3.10.3) for graphical visualization of communities and major connector hubs within the network, as well as for mapping the proteins previously annotated as ‘neuronal’.

## Results

### Proteomic analysis

In order to identify changes in protein levels during the initial stages of *Ciona* metamorphosis, protein extracts from larvae (200 pooled individuals) at three different developmental stages, Swimming (SwL, stage 28 of Hotta), Settled (SetL, stage 32 of Hotta) and Metamorphosis (MetL, stage 34 of Hotta), were analyzed by liquid chromatography mass spectrometry (LC-ESI-MS/MS). For each stage, three biological replicates were analyzed. After performing quality control on the obtained results, a sample from the SetL stage was discarded due to low or absent signal, likely resulting from technical issues during protein extraction.

**Fig 1:**
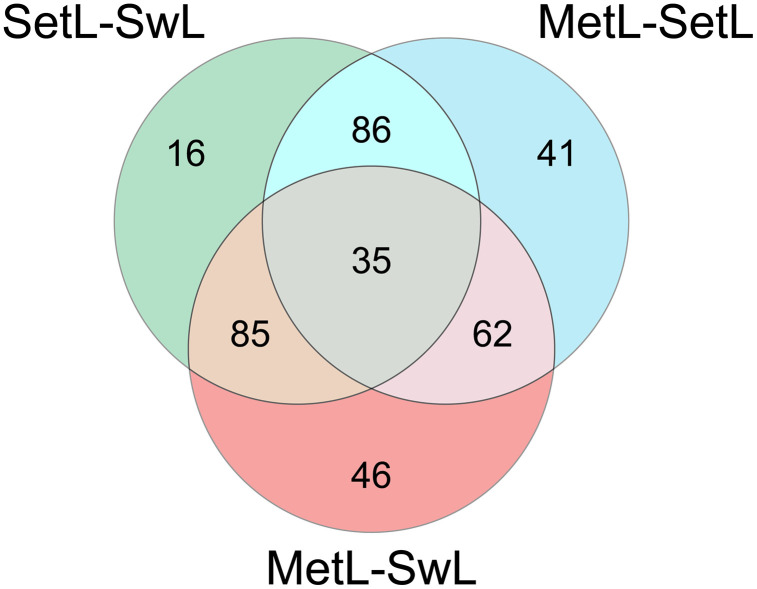
Venn diagram showing the distribution of differentially abundant proteins across the three experimental comparisons. The overlaps represent shared proteins among the comparisons SetL-SwL, MetL-SetL, and MetL-SwL, while unique regions indicate proteins specifically varying in a single comparison. Numbers within each section correspond to the count of proteins in that category.

Overall, the analysis led to the identification of 1152 proteins. Among them, 405 are differentially expressed (DEPs), either up or downregulated, in at least one stage comparison (ANOVA and Tukey’s test, p-value < 0.05) (S1 Table). The number of significantly upregulated proteins was 70, 124 and 74 for the SetL versus SwL, MetL versus SetL and MetL versus SwL comparisons, respectively; while 152, 100 and 154 proteins resulted downregulated in the same comparisons (Table 1). A total of 35 proteins, out of 405, were found to be differentially modulated across all the stage comparisons (Fig 1).

**Table 1:** Number of overexpressed and underexpressed proteins in the three stages comparisons.

	Overexpressed	Underexpressed	Total
**SetL vs. SwL**	70	152	222
**MetL vs. SetL**	124	100	224
**MetL vs. SwL**	74	154	228

### Annotation of DE proteins

DEPs were then identified integrating the current UniProtKb annotation of *Ciona* proteome (UP000008144) and the STRING annotation (S1 Table). 283 proteins were fully annotated with the corresponding protein name and associated molecular function/activity, while other 99 were classified based upon the presence of a specific domain (referred to as ‘domain-containing protein’). The most represented domains are VWFA domain, EF-hand domain, RRM domain, IF rod domain and Sushi domain. Finally, 23 proteins remain completely uncharacterized (‘uncharacterized protein’). The annotation of the top 10 overexpressed and top 10 underexpressed proteins in the three stage comparisons is shown in Tables 2–4, respectively. Notably, several annotated DEPs displayed an opposite regulation when comparing the transition between the SwL and SetL stage towards the transition between the SetL and MetL stage, with 32 of them being upregulated going from the first to the second stage and downregulated from the second to the third stage, while 70 showing an inverse trend (see Table 5, S1 Table).

**Table 2:** List of the top 10 overexpressed and 10 underexpressed proteins in the SetL-SwL comparison.

Rank	UniprotKB ID	Protein name^a^	Gene name^b^	Log2FC SetL/SwL
**Overexpressed**				
1	F6Q7V3	VWFA domain-containing protein	na	3.718
2	F6YF25	VWFA domain-containing protein	na	1.949
3	F6R9M9	EF-hand domain-containing protein	na	1.938
4	H2Y0U3	Uncharacterized protein	na	1.934
5	F6V1M6	Uncharacterized protein	meta2	1.868
6	H2XLP7	VWFA domain-containing protein	na	1.757
7	F6QFI4	Signal peptidase complex subunit 2	LOC100178515	1.626
8	F6R1X5	Cell division cycle 42	cdc42	1.619
9	F6XQP8	ZnF_CDGSH domain-containing protein	LOC100179954	1.434
10	F6R9G4	EF-hand domain-containing protein	na	1.424
**Underexpressed**				
1	F6QHG3	Uncharacterized protein	na	−3.006
2	F6YX55	VWFA domain-containing protein	na	−2.272
3	F6U9F4	Cytoplasmic aconitate hydratase	LOC100186938	−2.174
4	H2Y1H9	Protein transport protein SEC23	LOC100183335	−1.917
5	F7AJ84	Glucosidase II subunit alpha	na	−1.770
6	F6WUH0	NADH dehydrogenase [ubiquinone] iron-sulfur protein 3, mitochondrial (Complex I-30kD)	LOC100183052	−1.696
7	F7AKV1	Villin-1	LOC100185031	−1.681
8	F6X0F3	Microtubule-associated protein RP/EB family member 1	LOC100181072	−1.666
9	F6PTZ3	Endothelin-converting enzyme 1	na	−1.598
10	F6U8C7	Eukaryotic translation initiation factor 3 subunit B (eIF3b)	LOC100182767	−1.595

^a^ UniProtKB annotation – release 2024_04

^b^ NCBI Gene

**Table 3:** List of the top 10 overexpressed and 10 underexpressed proteins in the MetL-SetL comparison.

Rank	UniprotKB ID	Protein name^a^	Gene name^b^	Log2FC MetL/SetL
**Overexpressed**				
1	H2Y3J3	Uncharacterized LOC100186633	LOC100186633	2.030
2	F6Q6Q9	IF rod domain-containing protein	LOC100175966	1.994
3	F7BL29	IF rod domain-containing protein	LOC100182102	1.948
4	F7AEC5	Beta-catenin	na	1.924
5	F6WUH0	NADH dehydrogenase [ubiquinone] iron-sulfur protein 3, mitochondrial (Complex I-30kD)	LOC100183052	1.808
6	F6XE85	Cytokine-like nuclear factor N-PAC (Glyoxylate reductase 1 homolog)	LOC100180692	1.796
7	H2XRE2	Endoplasmin (Heat shock protein 90 kDa beta member 1)	na	1.711
8	F6U8C7	Eukaryotic translation initiation factor 3 subunit B (eIF3b)	LOC100182767	1.705
9	F7AKV1	Villin-1	LOC100185031	1.585
10	H2XK79	ADP/ATP translocase (ADP,ATP carrier protein)	na	1.514
**Underexpressed**				
1	F6VNG2	Annexin	na	−2.594
2	F6UGK1	IF rod domain-containing protein	na	−2.262
3	F6ZX95	VWFA domain-containing protein	na	−2.213
4	F6PID9	Lipoxygenase domain-containing protein	na	−1.948
5	F7ACZ6	Uncharacterized protein	na	−1.947
6	F6XQP8	CDGSH iron-sulfur domain-containing protein 2 homologue	LOC100179954	−1.737
7	F6VFA0	Large ribosomal subunit protein uL29 (60S ribosomal protein L35)	LOC100179536	−1.726
8	F6PPN6	Uromodulin	LOC100186455	−1.713
9	H2XRC1	Uncharacterized protein	na	−1.709
10	F6Y5B7	E2 ubiquitin-conjugating enzyme (EC 2.3.2.23)	LOC100185831	−1.676

^a^ UniProtKB annotation – release 2024_04

^b^ NCBI Gene

**Table 4:** List of the top 10 overexpressed and 10 underexpressed proteins in the MetL-SwL comparison.

Rank	UniprotKB ID	Protein name^a^	Gene name^b^	Log2FC MetL/SwL
**Overexpressed**				
1	F6Q7V3	VWFA domain-containing protein	na	3.270
2	F7BL29	IF rod domain-containing protein	LOC100182102	2.589
3	F6V1M6	Uncharacterized protein	meta2	2.419
4	H2Y0U3	Uncharacterized protein	na	2.147
5	F6Q6Q9	IF rod domain-containing protein	LOC100175966	2.131
6	F6R9M9	EF-hand domain-containing protein	na	2.120
7	F7BH30	Creatine Kinase	LOC100176946	2.113
8	F6R9G4	EF-hand domain-containing protein	na	1.897
9	H2XTV2	Troponin I	LOC100179388	1.865
10	F7AEC5	Beta-catenin	na	1.738
**Underexpressed**				
1	F6QHG3	Uncharacterized protein	na	−3.681
2	F6VNG2	Annexin	na	−3.256
3	F6UGK1	IF rod domain-containing protein	na	−2.947
4	F6YX55	VWFA domain-containing protein	na	−2.819
5	F6ZX95	VWFA domain-containing protein	na	−2.747
6	F6PTZ3	Endothelin-converting enzyme 1	na	−2.746
7	F6UJG6	Lipoxygenase domain-containing protein	na	−2.417
8	F6WYH0	Integrin beta	LOC100182056	−2.405
9	H2XRC1	Uncharacterized protein	na	−2.302
10	F6R0J7	Metalloendopeptidase	na	−2.152

^a^ UniProtKB annotation – release 2024_04

^b^ NCBI Gene

**Table 5:** List of the most significant proteins displaying opposite regulation in the SetL-SwL and MetL-SetL comparisons.

UniprotKB ID	Protein name^a^	Log2FC SetL/SwL	Log2FC MetL/SetL
**Upregulated in SetL vs. SwL and downregulated in MetL vs. SetL**			
F6XQP8	CDGSH iron-sulfur domain-containing protein 2 homologue	1.434	−1.737
F6Y5B7	E2 ubiquitin-conjugating enzyme (EC 2.3.2.23)	1.246	−1.676
F6R1X5	Cell division cycle 42	1.619	−1.252
F6QFI4	Signal peptidase complex subunit 2	1.626	−1.149
H2XUQ6	Hyaluronan/mRNA-binding protein domain-containing protein	1.390	−1.386
F6Z215	Mitochondrial cytochrome c oxidase subunit VIc/VIIs domain-containing protein	1.165	−1.516
F6TJY9	40S ribosomal protein S21	1.371	−1.156
H2XR12	GP-PDE domain-containing protein	1.140	−1.234
F6Y4V4	E2 ubiquitin-conjugating enzyme (EC 2.3.2.23)	0.931	−1.404
A0A1W2WF05	Ubiquitin-like-conjugating enzyme ATG3 (Autophagy-related protein 3)	1.092	−1.181
F6SVM4	Eukaryotic translation initiation factor 3 subunit K (eIF3k) (eIF-3 p25)	1.336	−0.928
F6ZBX3	Peptidase S1 domain-containing protein	0.751	−1.448
A0A1W3JRI0	Ubiquitin-like domain-containing protein (r27a)	1.105	−1.061
F6T2M3	Thrombospondin	1.376	−0.730
F6PXX9	Prosaposin	0.906	−1.143
F7A5D0	Nucleolin like protein CiRGG1	1.219	−0.790
F6U5F1	ATP citrate synthase (EC 2.3.3.8)	0.852	−1.155
F6Q5B7	Plasminogen receptor (KT)	0.899	−1.003
F6TJ76	Proteasome subunit beta	0.730	−1.162
F6TQ69	Importin-7	0.782	−1.056
**Downregulated in SetL vs. SwL and upregulated in MetL vs. SetL**			
F6S4Q0	Acetyl-CoA acetyltransferase, mitochondrial-like	−1.092	0.917
F6W1H2	Coatomer subunit alpha	−1.300	0.821
F6RID7	Triosephosphate isomerase (EC 5.3.1.1)	−1.325	0.811
H2XQC1	PX domain-containing protein	−1.194	1.041
A0A1W2WP01	Dolichyl-diphosphooligosaccharide--protein glycosyltransferase subunit DAD1 (Oligosaccharyl transferase subunit DAD1)	−1.207	1.028
F7ANT6	Cytochrome c oxidase subunit 5A, mitochondrial (Cytochrome c oxidase polypeptide Va)	−1.108	1.216
A0A1W2WJ91	Large ribosomal subunit protein eL43 (60S ribosomal protein L37a)	−1.440	0.913
H2XK79	ADP/ATP translocase (ADP,ATP carrier protein)	−1.190	1.514
F6X0F3	Microtubule-associated protein RP/EB family member 1	−1.666	1.049
A0A1W2WCG1	Large ribosomal subunit protein eL22 (60S ribosomal protein L22)	−1.416	1.353
F6XE85	Cytokine-like nuclear factor N-PAC (Glyoxylate reductase 1 homolog) (Nuclear protein NP60) (Putative oxidoreductase GLYR1)	−1.068	1.796
F7B898	RNA helicase (EC 3.6.4.13) (DEAD box protein 6)	−1.563	1.423
F7AJ84	Glucosidase II subunit alpha	−1.770	1.265
H2XRE2	Endoplasmin (Heat shock protein 90 kDa beta member 1)	−1.393	1.711
H2Y1H9	Protein transport protein SEC23	−1.917	1.226
F7AKV1	Villin-1	−1.681	1.585
F6U8C7	Eukaryotic translation initiation factor 3 subunit B (eIF3b) (Eukaryotic translation initiation factor 3 subunit 9)	−1.595	1.705
H2Y3J3	Uncharacterized LOC100186633	−1.351	2.030
F6U9F4	Cytoplasmic aconitate hydratase	−2.174	1.219
F6WUH0	NADH dehydrogenase [ubiquinone] iron-sulfur protein 3, mitochondrial (Complex I-30kD) (NADH-ubiquinone oxidoreductase 30 kDa subunit)	−1.696	1.808

^a^UniProtKB annotation – release 2024_04.

#### Identification of neuronal DEPs.

To explore the involvement of the identified DEPs in neuronal processes and, potentially, in neurodegeneration, we first incorporated an additional layer of annotation by cross-referencing DEPs with annotations indicating relevance to the nervous system and neuronal functions, as supported by multiple data annotation sources, and we identified 41 neuronal DEPs (S2 Table). Secondly, to determine whether the DEPs are present in the nervous system of *Ciona*, we additionally analyzed the dataset published by Cao et al., which provides a single-cell transcriptomic analysis of the larval nervous system [23]. Notably, this analysis revealed that more than 50% of the genes encoding the DEPs we identified (216/405) are expressed in at least one neuronal subtype (neuronal subcluster) of the swimming larva (S2 Table), suggesting that these DEPs may play important roles in nervous tissue remodeling processes. Altogether, 233 DEPs could be identified as relevant for neuronal processes (S2 Table).

### Enrichment analysis

**Fig 2:**
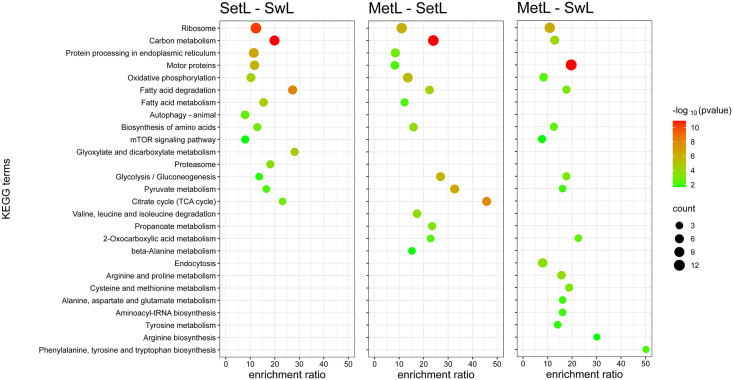
KEGG terms enrichment analysis of differentially expressed proteins across the experimental comparisons. The dot plots show enriched KEGG terms for the comparisons SetL vs. SwL, MetL vs. SetL, and MetL vs. SwL. The x-axis indicates the enrichment ratio, while the y-axis lists the enriched KEGG terms. The color gradient represents the statistical significance (−log₁₀ p-value), with red indicating higher significance, and the dot size corresponds to the number of proteins associated with each term.

To gain insights into the pathways and biological processes that are dynamically regulated during the metamorphosis, we carried out a KEGG pathway and Gene Ontology enrichment analysis on DEPs (Fig 2, S1 and S2 Figs; S3–S5 Tables). The KEGG database contains 13713 *Ciona* proteins, 7744 of which with a KEGG annotation. In our analysis, 75/222 SetL-SwL, 73/224 MetL-SetL and 69/228 MetL-SwL DEPs were annotated with at least one KEGG term, respectively. The analysis revealed enriched KEGG terms that were common to all stage comparisons, including ‘Motor proteins’, ‘Oxidative phosphorylation’, ‘Fatty acid degradation’ and ‘Biosynthesis of amino acids’. On the contrary, some pathways were found to be specifically enriched only in one comparison. Notably, ‘Autophagy pathway’ (comprising an autophagy-related gene and mitogen-activated protein kinases), ‘mTOR signaling pathway’, ‘Proteasome pathway’ (including proteasome and proteasome regulatory subunits) and ‘Glyoxylate and dicarboxylate metabolism’ were significantly enriched during the settlement of the swimming larva (SetL vs SwL). An enrichment in ‘Citrate cycle (TCA cycle)’, ‘Valine, leucine and isoleucine degradation’, ‘Propanoate metabolism and 2-Oxocarboxylic acid metabolism’ was found during the transition between the settled (SetL) and the metamorphosing (MetL) larva. Finally, pathways related to the metabolism and biosynthesis of some amino acids (particularly arginine, phenylalanine, tyrosine and tryptophan) and ‘Endocytosis’ (including proteins involved in the actin cytoskeleton organization) were enriched going from the SwL to the MetL stage (Fig 2).

Gene Ontology (GO) annotation covers approximately 44% (Biological Process, BP) and 47% (Molecular Function, MF) of *Ciona* proteins. We found 140/222, 139/224 and 148/228 DEPs associated with at least one GO-BP term and 168/222, 164/224, and 175/228 DEPs associated with at least one GO-MF term for the SetL-SwL, MetL-SetL and MetL-SwL comparison, respectively. GO enrichment analysis showed an involvement of fatty acid and lipid modification, as well as RNA/mRNA binding, for the swimming to settled larva transition. The switch between the SetL and MetL stage, on the other hand, was enriched in GO terms associated to nucleotide and carbohydrate metabolism/catabolism, tricarboxylic acid cycle, and regulation of translation. An enrichment in biological processes and molecular functions related to cytoskeleton organization (particularly actin cytoskeleton) was evidenced for the MetL vs SwL comparison (S1 and S2 Figs).

### Network analysis

**Fig 3:**
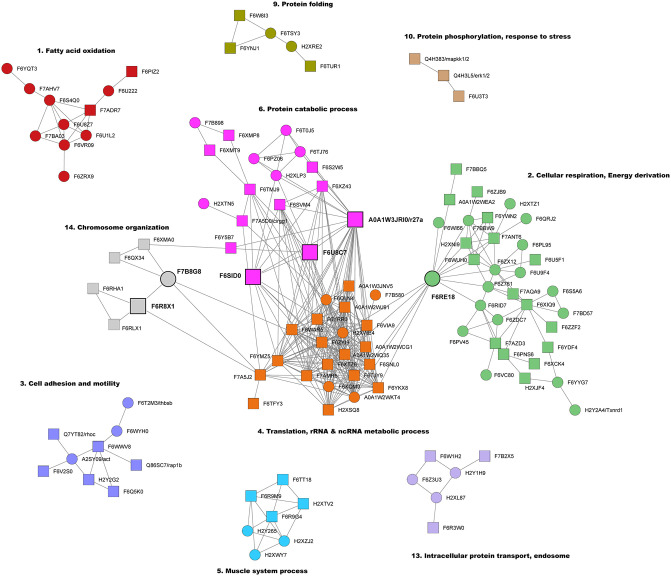
Network analysis of differentially expressed proteins in the SetL-SwL comparison. Node colors correspond to functional clusters (communities), and lines represent connections between proteins. Square nodes represent proteins annotated as ‘neuronal’, by combining STRING database and Cao et al., 2019 dataset (see S2 Table). The main connector hubs are represented by larger circles/squares (see Table 6, S5 Fig). Major clusters are annotated with the most representative biological process names, highlighting key functional modules.

To further uncover relevant functional modules and hub proteins involved in the metamorphosis of *Ciona*, we carried out a protein-protein interaction (PPI) network analysis. For each comparison, we defined a network among DEPs using the PPI from the STRING database (https://string-db.org/) [22]. Then, we identified network communities (clusters of proteins that exhibit a tendency to interact with one another) and obtained the so-called functional cartography [27], a classification of proteins into roles according to their pattern of intra- and inter-community interactions. This method allowed us to distinguish between hub proteins and non-hubs proteins of different kinds, and their relationships in each comparison (Fig 3, S3–S5 Figs, Table 6, S6–S11 Tables).

**Table 6:** List of connector hub proteins identified by PPI Network Analysis.

UniprotKB ID	Protein name^a^	Gene name^b^	Community	P	z-score
**SetL vs. SwL**					
F6RE18	ATP synthase subunit gamma	na	2	0.459184	1.7708472
F6SID0	Eukaryotic translation initiation factor 3 subunit E (eIF3e)	na	6	0.500000	0.9369872
F6U8C7	Eukaryotic translation initiation factor 3 subunit B (eIF3b)	LOC100182767	6	0.568047	0.9369872
A0A1W3JRI0	Ubiquitin-ribosomal protein eS31 fusion protein	r27a	6	0.444444	2.6137011
F6R8X1	RuvB-like helicase	LOC100183367	14	0.375000	1.2909944
F7B8G8	T-complex protein 1 subunit eta	LOC100185811	14	0.480000	1.2909944
**MetL vs. SetL**					
F7AZD3	Glucose-6-phosphate isomerase	na	8	0.345679	2.0579830
F6VYC6	Tyrosine--tRNA ligase	LOC100178583	8	0.500000	1.0289915
**MetL vs. SwL**					
F6PNS6	S-adenosylmethionine synthase	na	3	0.480000	0.7509393
F6VYC6	Tyrosine--tRNA ligase	LOC100178583	3	0.580000	1.8022542
F6RE18	ATP synthase subunit gamma	na	5	0.593750	1.8770039
F6SHZ7	F-actin-capping protein subunit beta	capzb	9	0.320000	1.5406578
F7A429	Transketolase	na	16	0.406250	0.7371293

^a^ UniProtKB annotation – release 2024_04, ^b^ NCBI Gene

P = participation coefficient, z-score = within-module degree score

For the transition between the SwL and SetL stage, a total of 15 modules (communities) were identified, 10 of which contained at least three proteins (Fig 3). Notably, four of these clusters are interconnected through six hub proteins (‘connector hubs’) (Table 6, S5 Fig). Modules 4, enriched for translation regulation and rRNA metabolic process, and 6, related to protein catabolic process, are tightly connected through three neuronal DEPs, specifically two subunits of Eukaryotic translation initiation factor (eIF3b and eIF3e) and the Ubiquitin-ribosomal protein eS31 fusion protein (r27a). These two modules are also linked to module 14 (chromosome organization) thanks to RuvB-like helicase and T-complex protein 1 subunit eta. Proteins belonging to Module 2, involved in cellular respiration, energy derivation and metabolism of nucleotides, and 4 (translation) are connected by ATP synthase subunit gamma. Modules 3 (cell adhesion and motility), 4 (translation), 6 (protein catabolic process), 10 (protein phosphorylation and response to stress) and 14 (chromosome organization) are particularly enriched in neuronal proteins (Fig 3). The MetL-SetL network is characterized by 19 clusters, 12 with at least three proteins. Seven of these modules are interconnected, with two hub proteins, Glucose-6-phosphate isomerase and Tyrosine-tRNA ligase, linking carbohydrate metabolism (Module 8) to fatty acid oxidation and cellular respiration processes (modules 2 and 19), on the one hand, and to translation, rRNA processing, protein folding and stabilization (Modules 3, 5 and 12), on the other hand (S3 and S5 Figs, Table 6). Module 1 is enriched with proteins involved in organismal development and neurogenesis. Finally, by analyzing the whole metamorphosis process, from SwL to MetL stage, we identified 19 modules (16 containing at least three proteins). 8 modules are interconnected through the interaction of five connector hub proteins (S4 and S5 Figs, Table 6). Central to this network is the Transketolase enzyme (annotated as neuronal DEP), bridging together carbohydrate metabolism (Module 16), tRNA metabolic process (Module 3, hub proteins S-adenosylmethionine synthase and Tyrosine--tRNA ligase), cellular respiration (Module 5, hub protein ATP synthase subunit gamma) and actin cytoskeleton organization (Module 9, hub protein F-actin-capping protein subunit beta). Of note, Modules 1, related to cell adhesion and neurogenesis, 3 (tRNA metabolic process), 6 (translation) and 9 (actin cytoskeleton organization) contain several proteins annotated as ‘neuronal’.

## Discussion

Proteomic analysis has already proved to be a successful approach to quantify proteins in unfertilized eggs and to track proteomic changes throughout the embryogenesis of the ascidian *Ciona intestinalis* [28]. We used the same approach during the metamorphosis of *Ciona* and identified 1152 proteins. Among them, 405 were differentially expressed in at least one stage comparison and could represent important tools to delineate the changes at cellular level occurring during the first stages of post-larval development. Most of the differentially expressed proteins (DEPs) were identified and associated with their function, using the most updated proteome annotation of *Ciona*.

A validation of our results was the identification of the Meta-2 protein among the top ten upregulated ones in the comparison between swimming larvae and settled/metamorphosing larvae (Tables 2 and 4). This observation is consistent with a transcriptome analysis in *Ciona*, which reported that *Ci-meta2* expression begins at the larval stage and is upregulated in metamorphosing juveniles [29]. *Ci-meta2* expression was detected in the adhesive organs, structures involved in settlement, as well as in the neck region of the central nervous system and in the dorsal epidermis of the trunk corresponding to the siphon primordium. It has been suggested that Meta-2 may play a role in the dynamic rearrangement of cells during ascidian metamorphosis [29], even though a functional analysis of this protein has not been carried out.

Interestingly, several DEPs are annotated as containing domains characteristic of proteins involved in cellular processes that play key roles during development, such as cell adhesion (VWFA), calcium signaling (EF-Hand) and cytoskeleton assemblage (IF rod). Other DEPs were identified as Sushi domain-containing proteins. *Ci-sushi* gene (specifically KH.C11.274 or p-selectin) in *Ciona* plays a crucial role in the metamorphic tail regression of the larva. It codifies for a cell-cell communication protein containing “sushi domains” (complement control protein modules) that is up-regulated during the transition from a free-swimming larva to a sessile juvenile [12].

Our analysis revealed that numerous DEPs are specifically upregulated or downregulated in the settled larva when compared with both the swimming larva and the metamorphosed juveniles. These patterns point to a unique proteomic landscape characterizing the settled stage, in which substantial molecular remodeling occurs and critical pathways are differentially regulated relative to adjacent developmental stages.

We observed that several proteins with this expression profile are involved in autophagy, ubiquitination, and proteasome regulation. Among these, r27a resulted also a hub protein in network analysis linking functional modules of catabolic processes and those of translation, rRNA and scRNA metabolic processes. It is well known that autophagy is a crucial process for embryonic and postnatal development, contributing to cell fate decisions by removing components that are no longer needed, which is critical for proper differentiation. mTOR (mammalian target of rapamycin) is a key actor of autophagic process and Gene Ontology enrichment analysis evidenced that its signaling pathway was significantly upregulated in SetL compared to both SwL and MetL stages. mTOR is a protein kinase that regulates cell growth, metabolism, proliferation, and survival [30]. It is a master regulator that integrates signals from nutrients, growth factors, and energy levels to control a cell’s growth and functions. Dysregulation of the mTOR pathway is implicated in diseases like cancer, diabetes, and aging [31,32].

Enrichment and network analyses both evidenced that the overall analyzed process, from swimming larva to settled larva, is characterized by differential expression of proteins involved in actin cytoskeleton organization. Noteworthy, the specific community (actin cytoskeleton organization) in network analysis contains several proteins such as RapB1, a GTP-binding protein, and RhoC, a small GTPase, that play a key role in cell migration, cytoskeletal organization, and cell division [33]. This is consistent with the events characterizing the early onset of metamorphosis, such as tail absorption, that is dependent on actin filaments [34–36].

Among DEPs detected by our analysis correlated with actin cytoskeleton, Cdc42, Capz and Villin-1 are of particular interest. Cdc42 (Cell division cycle 42) protein is among the top 10 overexpressed proteins in the transition from SwL to SetL stage, and it is subsequently downregulated from SetL to MetL (Tables 2 and 5). Cdc42 is a member of the Rho GTPase family; members of this family are regulators of F-actin polymerization [37], acting as molecular switches by cycling between an inactive GDP-bound state and an active GTP-bound state. Noteworthy, both alpha and beta subunits of Capz, another actin cytoskeleton interacting protein, are downregulated during *Ciona* metamorphosis and were found to be hub proteins in the MetL-SwL network (S4 Fig, Table 6, S1 Table). Capz is an actin capping protein that regulates actin filament growth by binding to the barbed ends of actin filaments, thereby preventing further polymerization [38]. Another protein interacting with actin cytoskeleton that is upregulated in SetL stage compared to previous and following stages is Villin-1. It is a major actin-modifying protein that is associated with microvillar actin filaments [39]. In *Homo*, it is expressed prevalently in renal and gastrointestinal epithelial cells. It has been demonstrated that Villin regulates epithelial cell morphology, actin reorganization, and cell motility [40].

Another cellular process known to play a critical role at the beginning of metamorphosis in ascidians is programmed cell death. In *C. intestinalis*, tail absorption is driven by apoptosis starting at the tail extremity and propagating along the tail. Apoptosis is caspase dependent, involves ERK activation and affects epidermal as well as muscle and notochord cells [13,41]. Among proteins known to be involved in apoptosis, we identified only ERK as differently expressed. It displayed its highest expression in the swimming larva and decreased in the settled and metamorphosis stages (S1 Table). Although not primarily involved in apoptosis, another interesting DEP is Adenine nucleotide translocase (ANT), a mitochondrial inner membrane protein known to have an anti-apoptotic function in specific cellular contexts [42,43]. It showed an opposite regulation, being downmodulated in the SwL to SetL transition and upregulated going from SetL to MetL (Table 5, S1 Table). The fact that we identified only a limited number of actors involved in apoptosis and a larger number associated with autophagy is consistent with the findings of Baghdiguian et al. [44], who reported a decrease in the number of TUNEL-positive cells in stage 1 juveniles compared to tadpoles, suggesting a predominant role of autophagy over apoptosis during the late developmental transition from larva to juvenile. On the other hand, it is also possible that our analysis was unable to detect differential expression levels of proteins involved in apoptosis, because their regulation occurs predominantly at the post-translational level, for example through proteolytic cleavage in the case of caspases or phosphorylation in the case of kinases.

Several other proteins involved in Citrate cycle (TCA cycle), Valine, leucine and isoleucine degradation, Propanoate metabolism and 2-Oxocarboxylic acid metabolism, were found overexpressed during the transition between the settled and the metamorphosing larva, indicating activation of metabolic processes necessary to rebuild the juvenile organs.

One of the aims of our study was the identification of novel factors potentially involved in neurodegeneration, leveraging the ascidian *Ciona*, which naturally undergoes a programmed loss of specific neurons as the swimming larva transitions into a sessile juvenile [10]. Our proteomic approach is well supported by prior studies, as proteomic analyses have proven powerful in revealing molecular changes underlying neurodegenerative disorders in humans [45]. Remarkably, several DEPs identified in our analysis have also been reported as dysregulated or impaired in patients with Alzheimer’s disease [45] or other neurodegenerative diseases, including Annexin, Cdc42, Glial fibrillary acidic protein, NADH dehydrogenase, Peptidyl-prolyl cis-trans isomerase and Transketolase, among others (some relevant examples are reported in Table 7) [46–64]. This observation suggests that common mechanisms may underlie pathological neurodegeneration in humans and physiological neurodegeneration in ascidians.

**Table 7:** Examples of *Ciona* DEPs whose human orthologue is involved in neurodegenerative diseases.

Protein name	Role in neurodegenerative diseases	References
Annexin	Anti-inflammatory role; role in the prevention and reduction of amyloid-induced toxicity.	Bartolome et al. 2020; Bedrood et al. 2009; Ries et al. 2016
Capz	Decreased levels are associated with neurites and growth cones defects.	Davis et al. 2009
Cell division cycle 42	Upregulated in AD with associated loss of dendritic spines; upregulated in FTLD.	Saraceno et al. 2018; Umbayev et al. 2023; Zhu et al. 2023
E2 ubiquitin-conjugating enzyme	Impaired function allows Aβ and tau to accumulate, forming plaques and tangles in AD; involved in early synaptic dysfunction.	Al Mamun et al. 2020
Fatty acid-binding protein	Involved in α-synuclein aggregation, neurotoxicity, and neuroinflammation in PD; involved in neuroinflammation in AD.	Chen et al. 2025; Kawahata and Fukunaga 2023
Glial fibrillary acidic protein	Upregulated and involved in astrogliosis in AD and other neurodegenerative diseases.	Leipp et al. 2024
NADH dehydrogenase	Decreased activity linked to mitochondrial impairment, oxidative stress and neuron loss in PD.	Keeney et al. 2006; Marella et al. 2009
Peptidyl-prolyl cis-trans isomerase	Reduced activity linked to Aβ and tau pathology and loss of synaptic plasticity in AD.	Lee et al. 2011; Xu et al. 2017
Prosaposin	Involved in lipid homeostasis, protective role in PD; mutations associated to PD.	He et al. 2023; Oji et al. 2020
Transketolase	Impaired TK activity reduces neurogenesis, contributing to cognitive dysfunction in AD and other neurodegenerative conditions.	Gibson et al. 2000; Vinh Quc Lu’O’Ng and Nguyen 2011

AD = Alzheimer’s Disease; FTLD = Frontotemporal Lobar Degeneration; PD = Parkinson’s Disease.

The already mentioned Cdc42 also plays a role in neurodegeneration. In humans, it participates in axonogenesis and contributes to the development and progression of neurodegenerative disorders [51]. Its dysregulation appears to be a common feature across conditions: in Alzheimer’s disease (AD), dendritic spine loss correlates with increased Cdc42 activity [52], while elevated levels are observed in the frontal cortex of frontotemporal lobar degeneration (FTLD) patients [50]. Additionally, the Cdc42 effector CIP4 accumulates with disease severity in Huntington’s disease (HD), interacting with Huntingtin and contributing to toxicity [65]. Another DEP, Capz, is essential for neurite outgrowth and growth cone morphology. Through its regulation of actin and interaction with tau, Capz supports neuronal structure and stability [49,66]. Together, Cdc42 and Capz have complementary roles in actin remodeling—Cdc42 promotes polymerization, while Capz stabilizes filaments—and their imbalance can lead to cytoskeletal defects, neurite dystrophy, and synaptic loss [49,52].

The ubiquitin-proteasome system (UPS) targets cellular proteins for degradation but is also involved in the pathogenesis of Alzheimer’s disease (AD). Current investigations have revealed that UPS components are associated with both the early and late stages of AD [53].

Transketolase (TK) acts as a hub protein linking modules identified in the comparison between SwL and MetL stages (S4 Fig, Table 6). Its deregulation is associated with neurodegeneration due to its central role in thiamine (vitamin B1) metabolism and the pentose phosphate pathway (PPP). TK supports the production of NADPH and ribose-5-phosphate, which are essential for redox balance and nucleotide biosynthesis [67]. Thiamine deficiency reduces TK activity, impairing the PPP and decreasing NADPH and ribose-5-phosphate levels, thereby weakening oxidative stress defense and neurogenesis. In humans, this impairment disrupts hippocampal neurogenesis, reduces progenitor cell proliferation, and alters neuronal differentiation, contributing to cognitive dysfunction in neurodegenerative disorders [63,64].

Importantly, our network analysis also identified different communities associating proteins with recognized function and unknown proteins. Several of these communities included proteins that have been recognized as relevant for neuronal function, based both on Gene Ontology and expression analysis in the larval nervous system [23] (Fig 3, S3 and S4 Figs, S2 Table). We think that further analysis of components of these communities could be promising to identify new actors in neurodegeneration.

Recent advances in ascidian metamorphosis research have uncovered its cellular dynamics at unprecedented resolution through the integration of single-cell omics, functional imaging, and genetic perturbation approaches [68–70]. In the context of these findings, our study further refines the understanding of the cellular and molecular mechanisms underlying metamorphosis, by deeply characterizing at network level differential protein expression and confirming that cytoskeletal remodeling, autophagy, and cell differentiation are central drivers of this process.

We are aware that our study has several limitations. First, we identified 1152 proteins, representing only a subset of the 7057 unique proteins reported by Frese et al. [28], that tracked proteomic changes throughout embryogenesis of *Ciona*, when at least half of the protein-coding genes are expressed. This broader developmental coverage captures a much wider proteomic landscape, as embryonic stages are characterized by a highly dynamic and stage-specific protein expression. In contrast, our analysis encompassed only three developmental larval stages spanning the metamorphosis process, which are expected to display a more limited proteome. Moreover, the two studies employed different quantitative strategies (label-free in our study vs Tandem Mass Tag (TMT)-based in Frese’s work) and the reference protein databases differed substantially. We used a publicly available reference resource, namely the UniProt reference proteome for *Ciona intestinalis* (UP000008144), which contains approximately one-tenth the number of entries compared to the more extensive (and partially redundant) KY21 dataset used in Frese et al [28]. The size and annotation depth of the search database directly influence the number of possible identifications, thereby affecting total protein counts and potentially limiting the comprehensive interpretation of the underlying biological processes. In addition, for one developmental stage, biological duplicates were used instead of triplicates. Although this reduces statistical power, each replicate consisted of a pooled sample of 200 whole larvae, which minimizes inter-individual variability and provides a robust estimate of the average proteomic profile. Taken together, differences in biological sampling (developmental breadth), quantitative strategy and reference database can collectively explain the discrepancy in total protein identifications. Therefore, the lower number of detected proteins in our study likely reflects differences in experimental design and analytical scope rather than an inherent limitation in method sensitivity.

Moreover, we analyzed the proteome of the entire organisms and we could have missed more subtle changes specifically occurring in the nervous system. Thus, it is expected that the proteins and the processes identified in this study have a role not only in neurons but also in non-neuronal cells. Furthermore, the incomplete functional annotation of proteins in this species hampers a comprehensive interpretation of the underlying biological processes. Nevertheless, the set of DEPs and processes identified here provides a valuable basis for further elucidating the main mechanisms involved in ascidian metamorphosis and neurodegeneration.

## Supporting information

S1 Fig Gene Ontology Biological Process (GOBP) terms enrichment analysis of differentially expressed proteins across the experimental comparisons. The dot plots show enriched GOBP terms for the comparisons SetL vs SwL, MetL vs SetL, and MetL vs SwL. The x-axis indicates the enrichment ratio, while the y-axis lists the enriched GOBP terms. The color gradient represents the statistical significance (−log₁₀ p-value), with red indicating higher significance, and the dot size corresponds to the number of proteins associated with each term.
(TIF)

S2 Fig Gene Ontology Molecular Function (GOMF) terms enrichment analysis of differentially expressed proteins across the experimental comparisons. The dot plots show enriched GOMF terms for the comparisons SetL vs SwL, MetL vs SetL, and MetL vs SwL. The x-axis indicates the enrichment ratio, while the y-axis lists the enriched GOMF terms. The color gradient represents the statistical significance (−log₁₀ p-value), with red indicating higher significance, and the dot size corresponds to the number of proteins associated with each term.
(TIF)

S3 Fig Network analysis of differentially expressed proteins in the MetL-SetL comparison. Node colors correspond to functional clusters (communities), and lines represent connections between proteins. Square nodes represent proteins annotated as ‘neuronal’, by combining STRING database and Cao et al., 2019 dataset (see S2 Table). The main connector hubs are represented by larger circles/squares (see Table 6, S5 Fig). Major clusters are annotated with the most representative biological process names, highlighting key functional modules.
(TIF)

S4 Fig Network analysis of differentially expressed proteins in the MetL-SwL comparison. Node colors correspond to functional clusters (communities), and lines represent connections between proteins. Square nodes represent proteins annotated as ‘neuronal’, by combining STRING database and Cao et al., 2019 dataset (see S2 Table). The main connector hubs are represented by larger circles/squares (see Table 6, S5 Fig). Major clusters are annotated with the most representative biological process names, highlighting key functional modules.
(TIF)

S5 Fig Functional cartography of the SetL-SwL, MetL-SetL and MetL-SwL networks. The maps show the distribution of proteins (nodes) within the network based on their participation coefficient (P, x-axis) and within-module degree z-score (z, y-axis). Nodes are categorized into seven distinct categories (R1 to R7) according to their functional connectivity profiles, each represented by a different color. This classification highlights the diversity of node roles in the network’s modular structure, distinguishing between nodes and hubs. The proteins classified as connector hubs (R6, pink area) in each network are labelled with the corresponding UniProtKB ID (corresponding to large circles/squares in Fig 3, S3 and S4 Figs). Squares represent proteins annotated as ‘neuronal’ (see S2 Table). Legend: R1 = Ultra-peripheral nodes: low z-score (low within-module connectivity) and very low participation coefficient (P), nodes with very few connections mostly limited to their own module; R2 = Peripheral nodes: low z-score and low P, nodes mostly connected within their own module but slightly more connected than R1; R3 = Connector nodes: low z-score and moderate P, nodes linking several modules but with limited within-module connections; R4 = Kinless nodes: low z-score and high P, nodes with connections distributed evenly across modules with no specific modular preference; R5 = Provincial hubs: high z-score (highly connected within their module) and low P, hubs with most links inside their module; R6 = Connector hubs: high z-score and moderate P, hubs connecting multiple modules, playing integrative network roles; R7 = Kinless hubs: high z-score and high P, hubs with extensive links across the whole network without module preference.
(TIFF)

S1 Table Annotated list of DEPs. Annotated list of differentially expressed proteins in at least one stage comparison.
(XLSX)

S2 Table List of DEPs annotated as neuronal. List of differentially expressed proteins annotated as involved in neuronal functions and/or expressed in swimming larva nervous system, obtained by combining STRING database and Cao et al., 2019 dataset.
(XLSX)

S3 Table SetL-SwL enrichment analysis. Gene Ontology and KEGG pathway enrichment analysis carried out on the differentially expressed proteins identified in the SetL-SwL comparison.
(XLSX)

S4 Table MetL-SetL enrichment analysis. Gene Ontology and KEGG pathway enrichment analysis carried out on the differentially expressed proteins identified in the MetL-SetL comparison.
(XLSX)

S5 Table MetL-SwL enrichment analysis. Gene Ontology and KEGG pathway enrichment analysis carried out on the differentially expressed proteins identified in the MetL-SwL comparison.
(XLSX)

S6 Table Functional cartography analysis performed on SetL-SwL network. (XLSX)

S7 Table Functional cartography analysis performed on MetL-SetL network. (XLSX)

S8 Table Functional cartography analysis performed on MetL-SwL network. (XLSX)

S9 Table Over representation analysis of the SetL-SwL network. GO biological process (BP) over representation analysis on protein communities identified in the SetL-SwL network.
(XLSX)

S10 Table Over representation analysis of the MetL-SetL network. GO biological process (BP) over representation analysis on protein communities identified in the MetL-SetL network.
(XLSX)

S11 Table Over representation analysis of the MetL-SwL network. GO biological process (BP) over representation analysis on protein communities identified in the MetL-SwL network.
(XLSX)
