# Supplementary figures and images for: Proteome analysis refines molecular processes underlying metamorphosis in the ascidian Ciona intestinalis
Source: PLoS One. 2026 Jun 1;21(6):e0350646. doi: 10.1371/journal.pone.0350646 (PMC13225635; doi:10.1371/journal.pone.0350646)

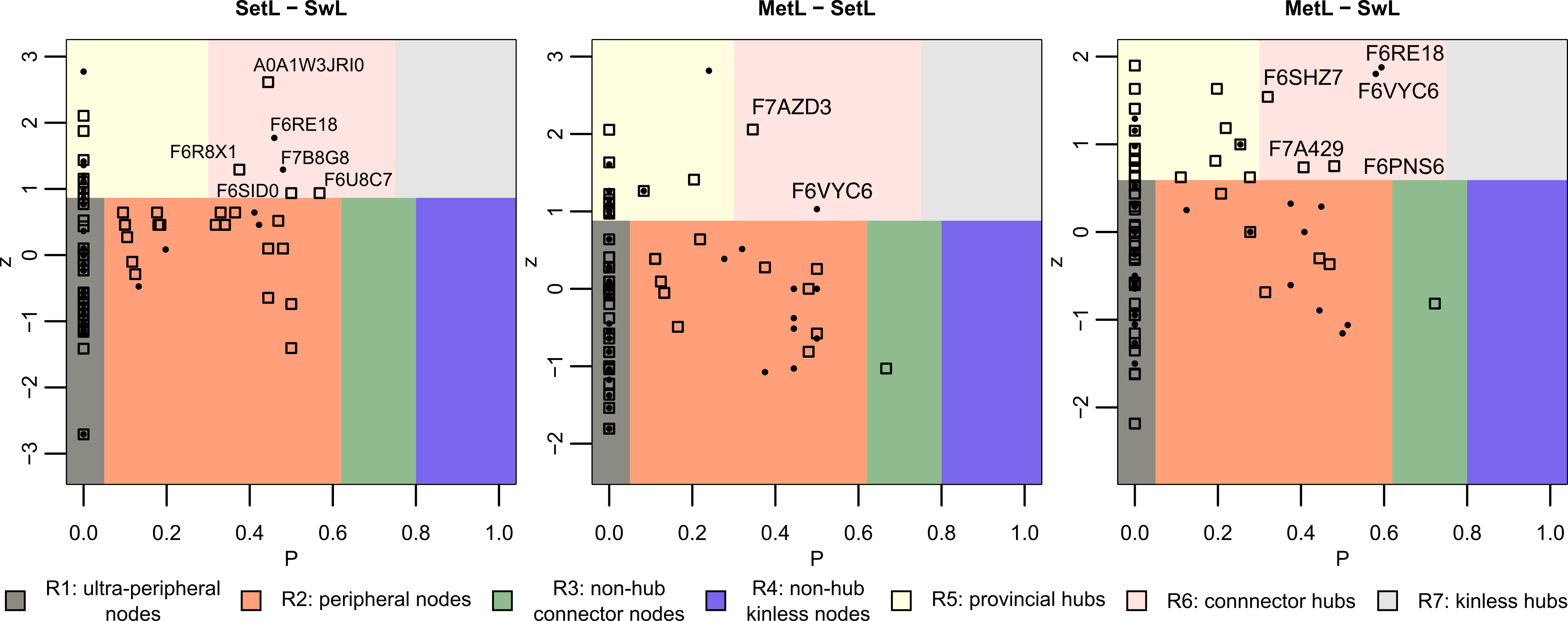

Supplement: S5 Fig — The maps show the distribution of proteins (nodes) within the network based on their participation coefficient (P, x-axis) and within-module degree z-score (z, y-axis). Nodes are categorized into seven distinct categories (R1 to R7) according to their functional connectivity profiles, each represented by a different color. This classification highlights the diversity of node roles in the network’s modular structure, distinguishing between nodes and hubs. The proteins classified as connector hubs (R6, pink area) in each network are labelled with the corresponding UniProtKB ID (corresponding to large circles/squares in Fig 3, S3 and S4 Figs). Squares represent proteins annotated as ‘neuronal’ (see S2 Table). Legend: R1 = Ultra-peripheral nodes: low z-score (low within-module connectivity) and very low participation coefficient (P), nodes with very few connections mostly limited to their own module; R2 = Peripheral nodes: low z-score and low P, nodes mostly connected within their own module but slightly more connected than R1; R3 = Connector nodes: low z-score and moderate P, nodes linking several modules but with limited within-module connections; R4 = Kinless nodes: low z-score and high P, nodes with connections distributed evenly across modules with no specific modular preference; R5 = Provincial hubs: high z-score (highly connected within their module) and low P, hubs with most links inside their module; R6 = Connector hubs: high z-score and moderate P, hubs connecting multiple modules, playing integrative network roles; R7 = Kinless hubs: high z-score and high P, hubs with extensive links across the whole network without module preference. (TIFF) [file pone.0350646.s005.tiff]
